# Resection of Diffuse Leiomyomas Related to Morcellation: A Rare Case Report

**DOI:** 10.1155/cris/1629800

**Published:** 2026-05-30

**Authors:** Sri Snehita Reddy Bonthu, Anish Jomy, William R. Salter, D. Rohan Jeyarajah

**Affiliations:** ^1^ Department of Surgery, Texas Christian University Anne Marie Burnett School of Medicine, 1100 W Rosedale Street, Fort Worth, 76104, Texas, USA; ^2^ Department of Surgery, Methodist Richardson Medical Center, 2831 E. President George Bush Highway, Richardson, 75082, Texas, USA; ^3^ Department of Obstetrics and Gynecology, Methodist Richardson Medical Center, 2831 E. President George Bush Highway, Richardson, 75082, Texas, USA

**Keywords:** abdominal wall endometriosis, benign metastasizing leiomyoma, case report, hepatic lobectomy

## Abstract

We report the case of a 42‐year‐old woman with a history of uterine fibroids treated with robotic myomectomy 12 years prior who presented with extensive uterine fibroids, perihepatic lesions, and an umbilical mass. Preoperative imaging and biopsy of perihepatic lesions confirmed benign leiomyomas with estrogen and progesterone receptor (PR) positivity. Multidisciplinary surgical management included total hysterectomy, partial hepatectomy, and abdominal wall resection with reconstruction. Histopathologic analysis confirmed the presence of uterine fibroids, hepatic benign metastasizing leiomyoma (BML), and abdominal wall endometriosis (AWE). This case highlights the rare phenomenon of concurrent BML and endometriosis and their potential association with prior uterine surgery involving morcellation.

## 1. Introduction

Benign metastasizing leiomyoma (BML) is a rare disorder that occurs in women of reproductive age in which histologically benign uterine smooth muscle tumors, commonly known as leiomyomas or fibroids, metastasize to extrauterine sites [1]. These sites include the lung and, less commonly, the heart, liver, esophagus, abdominal lymph nodes, skeletal muscle, skin, and central nervous system [2, 3]. BML typically affects premenopausal women with a history of leiomyomas that have undergone myomectomy or hysterectomy, indicating that the pathogenesis may involve iatrogenic spread during procedures such as morcellation [3, 4].

Abdominal wall endometriosis (AWE) is another condition that is associated with a history of gynecologic abdominal procedures like caesarean section, hysterectomy, or myomectomy, although spontaneous cases have also been reported. AWE is characterized by the proliferation of endometrial glands and stroma within the layers of the abdominal wall [5–9].

Here, we present an unusual case of BML with perihepatic and peritoneal involvement alongside AWE following prior robotic myomectomy requiring complex surgical management.

## 2. Case Presentation

A 42‐year‐old nulliparous woman presented for evaluation of a liver mass, progressively worsening abdominal distension and pain, and a growing mass over her umbilicus.

She had a long‐standing history of menorrhagia and irregular cycles secondary to uterine fibroids, for which she underwent robotic‐assisted laparoscopic myomectomy with power morcellation in 2013. The operative report from the robotic‐assisted laparoscopic myomectomy noted that fibroid pieces were morcellated and removed without mention of a containment method and did not document the presence of endometriosis during inspection of the pelvic or abdominal cavities. She remained asymptomatic until 2021, when she began experiencing recurrent menorrhagia and developing a bulge over her umbilical region.

Imaging from 2023 showed two heterogeneous enhancing soft tissue masses posterior to the right hepatic lobe, measuring 10 and 11 cm, exerting an extrinsic mass effect on the right hepatic lobe. She subsequently underwent a percutaneous tissue biopsy of the lesion, which showed a benign leiomyoma with positive immunostaining for estrogen receptor (ER), progesterone receptor (PR), desmin, and smooth muscle actin (SMA). The same imaging also identified a recurrently enlarged, fibroid‐laden uterus with the largest fibroid measuring 15 cm, a 4.4 cm subcutaneous left periumbilical mass, and a small umbilical hernia.

**Figure 1:**
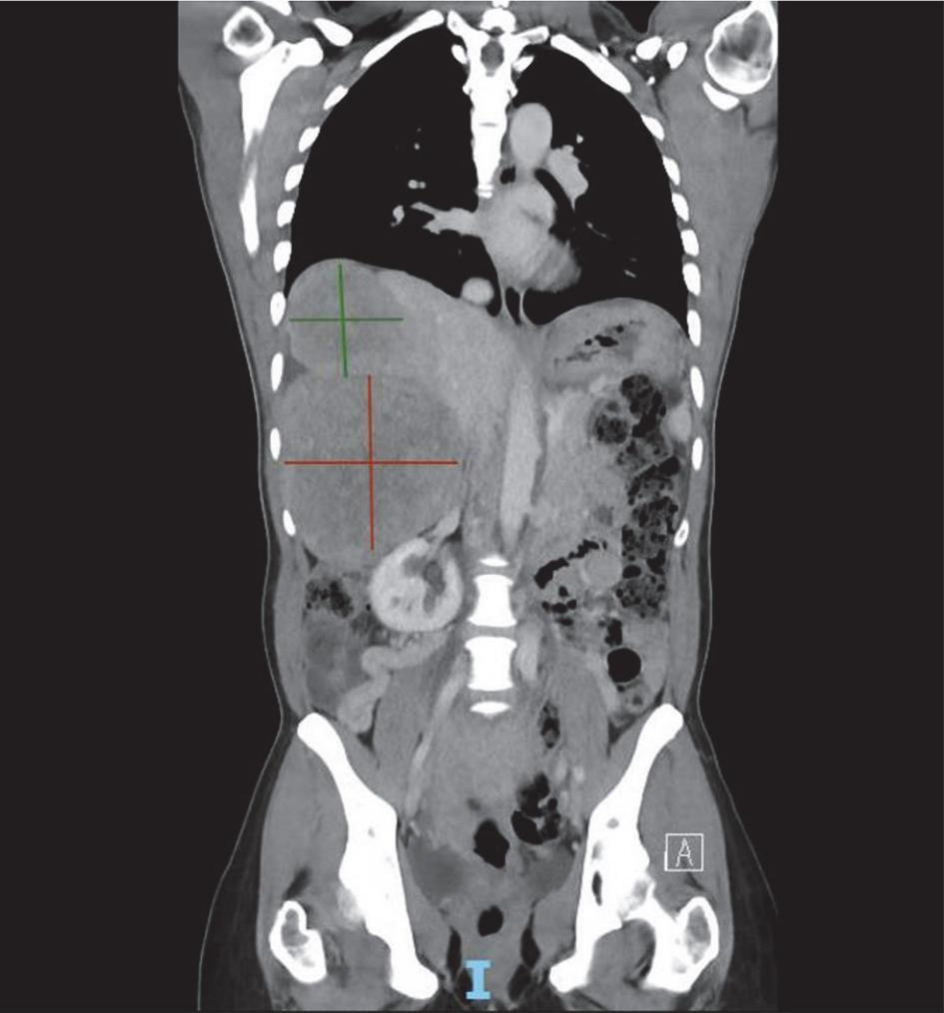
CT abdomen and pelvis coronal view showing superior hepatic mass (marked in green) and inferior hepatic mass (marked in red).

**Figure 2:**
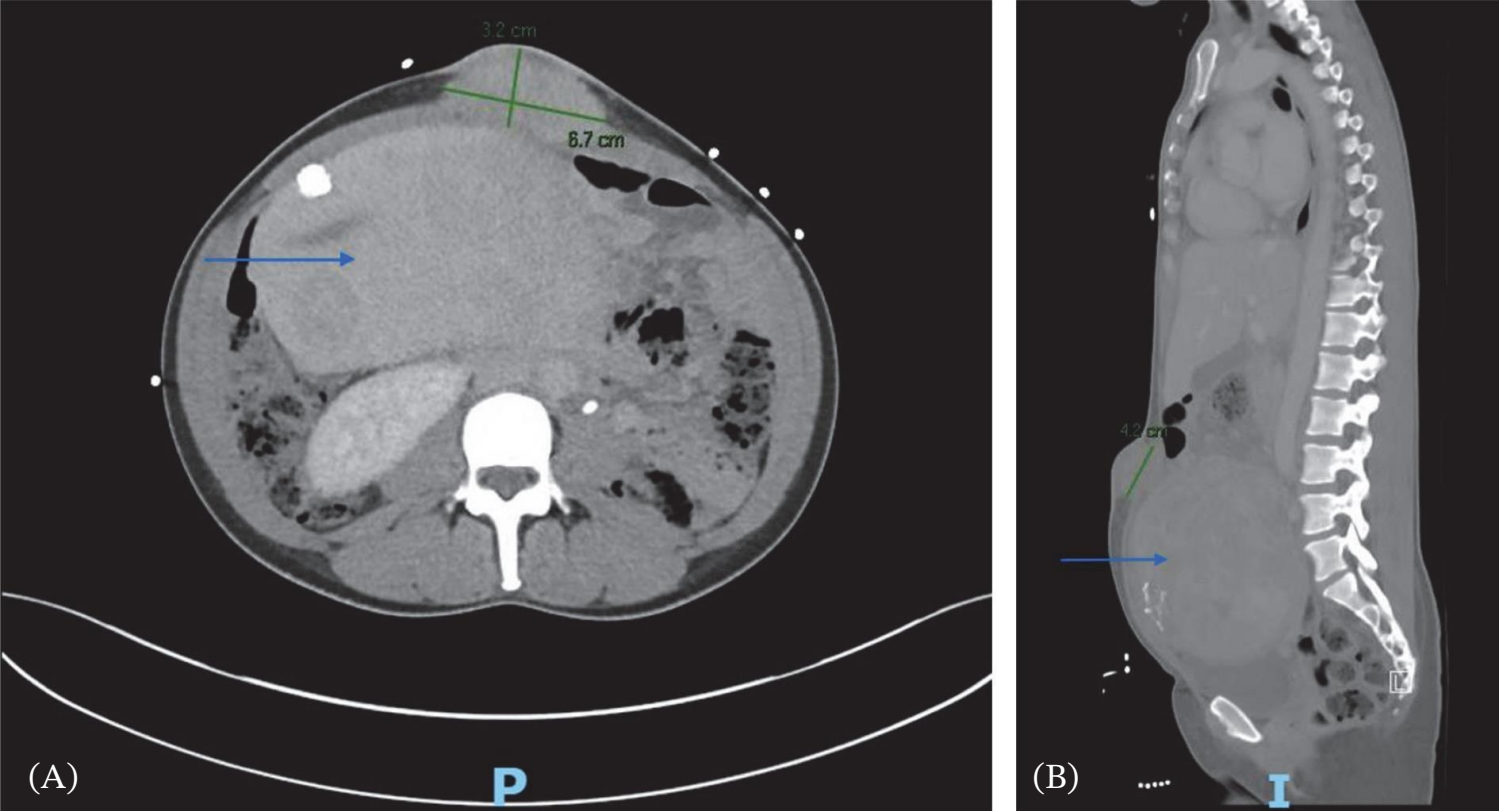
CT abdomen and pelvis depicting a ventral mass (marked in green) and a massively enlarged fibroid‐laden uterus (blue arrow) in (A) axial view and (B) sagittal view.

At the time of surgical oncology evaluation, she reported right‐sided abdominal pain and distension in addition to menorrhagia and irregular cycles, with menses lasting about 3 weeks and characterized by heavy bleeding. She did not report any family history of fibroids or malignancy. The physical exam was notable for hepatomegaly, abdominal tenderness in the right upper and lower quadrants, a lower abdominal mass arising from the pelvis, and a tender, hard, and immobile protruding umbilical mass. Computed tomography (CT) of the chest, abdomen, and pelvis was ordered to evaluate for interval changes compared with imaging performed 2 years prior. It revealed an increase in size of hepatic masses, now measuring 11.9 cm × 6.6 cm × 6.2 cm and 12.4 cm × 11 cm × 10.8 cm (Figure 1). The uterus was massively distended with multiple heterogeneous masses, and the soft tissue periumbilical mass also increased in size, measuring at 6.7 cm × 3.2 cm × 4.2 cm (Figure 2). Additionally, CT showed mild bilateral hydronephrosis, likely due to mass effect on the distal ureters by the enlarged uterus.

Based on the patient’s symptoms, imaging characteristics, and lesion size, the initial differential included leiomyosarcoma, desmoid tumor, gastrointestinal stromal tumor, and metastatic malignancy. Subsequent histopathologic evaluation and immunohistochemical staining favored a diagnosis of BML, with consideration of primary hepatic leiomyoma and uterine leiomyosarcoma. Given the size, symptomatic burden, and ER/PR‐positive status of her tumors, surgical resection of liver lesions and total hysterectomy were recommended. She was referred to obstetrics and gynecology (OB/GYN) for further operative planning.

**Figure 3:**
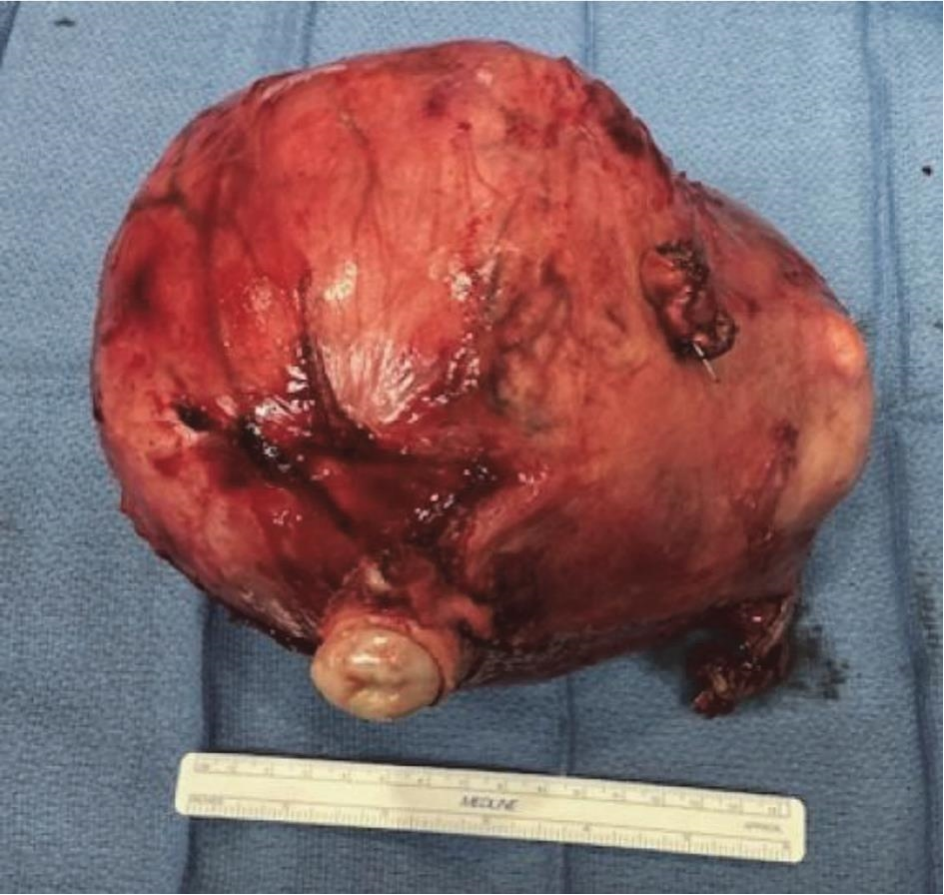
Gross specimen of the resected uterus with multiple intramural fibroids. The uterus is markedly enlarged and irregular in contour, consistent with leiomyomatous changes.

**Figure 4:**
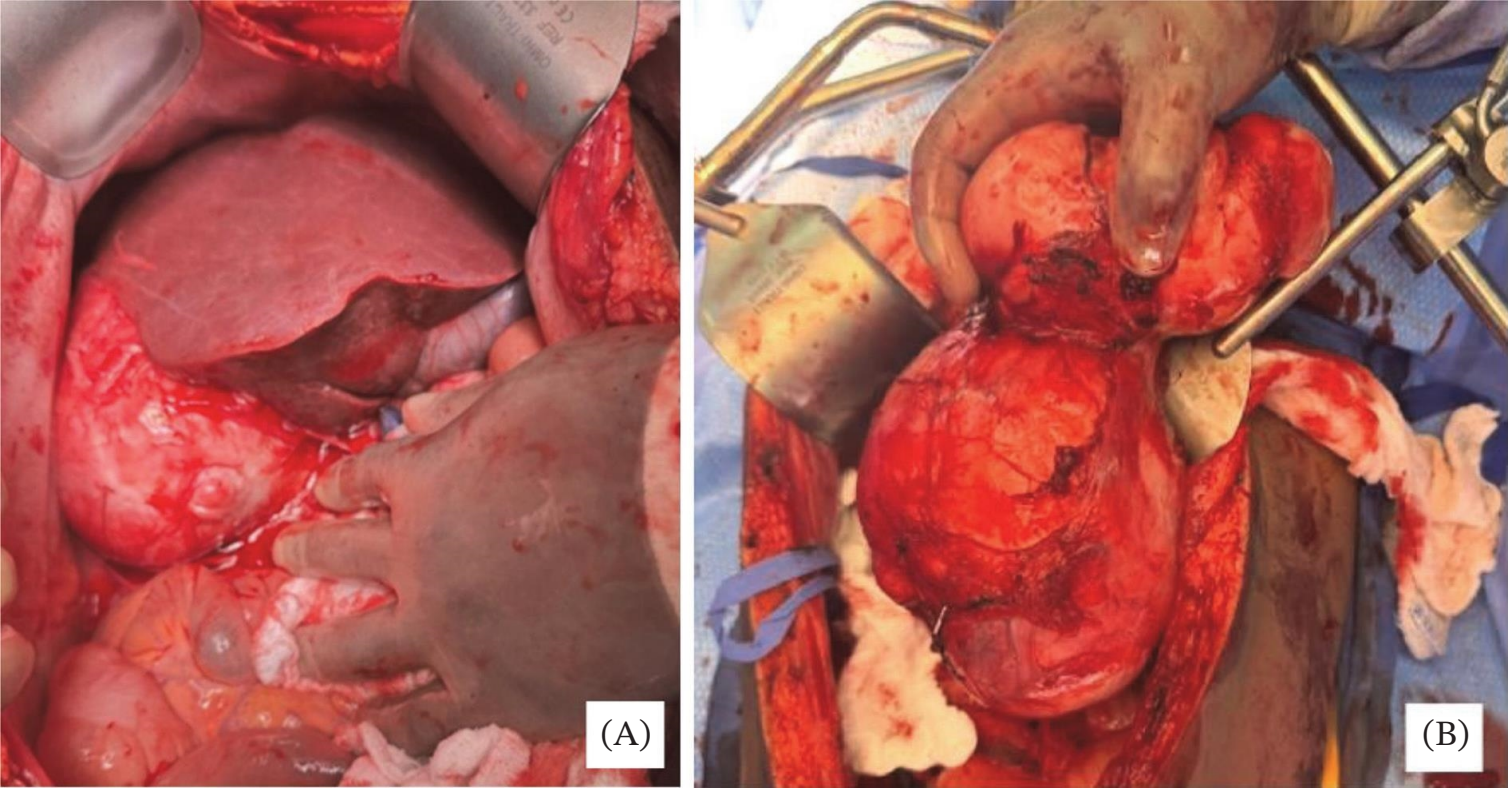
(A) Intraoperative image showing firm, well‐circumscribed leiomyomas located posterior to the right hepatic lobe. (B) Intraoperative image of two large, attached hepatic leiomyomas following resection.

The patient underwent a staged, multidisciplinary procedure. First, bilateral ureteral stents were placed by a urologist for bilateral hydronephrosis of the kidneys secondary to mass effect from the fibroid‐laden uterus revealed in the preoperative evaluation and for protection during pelvic dissection. Then, a midline laparotomy was performed. The 6 cm abdominal wall mass involving the umbilicus was resected en bloc with surrounding skin, fascia, and rectus muscle and sent to frozen section. Frozen section diagnosis revealed endometriosis. OB/GYN then performed a total abdominal hysterectomy and bilateral salpingectomy with preservation of the ovaries. The uterus was ~24 weeks gestational size with irregular contours consistent with fibroids (Figure 3). Bilateral fallopian tubes and ovaries appear to be normal in size, shape, and consistency without any obvious pathologic abnormalities. Surgical oncology then resected multiple small peritoneal nodules and mobilized the lesion of the right hepatic lobe. Lesions were partially adherent to the diaphragm, and mobilization resulted in partial diaphragmatic laceration and subsequent repair followed by pigtail chest tube placement into the right chest. The perihepatic tumors were successfully enucleated (Figure 4) and were followed by a partial right hepatic lobectomy. A pigtail chest tube was placed due to a small intraoperative pneumothorax.

Final pathology of perihepatic and peritoneal nodules confirmed the results of percutaneous biopsy of hepatic mass and supported the diagnosis of benign leiomyoma consistent with metastasizing uterine fibroid disease. On the other hand, final pathology of the abdominal wall mass frozen section revealed endometriosis. A tissue exam of uterine tissue revealed myometrium with leiomyomata with degenerative changes measuring up to 13 cm and the right fallopian tube involved by endometriosis with otherwise benign endometrium and cervix. The uterus and cervix weighed 2040 g. No malignancy was identified in any specimen.

The patient tolerated the procedure well and was transferred to recovery in stable condition. She is being followed postoperatively by surgical oncology and OB/GYN. She is expected to have significant symptom relief following tumor debulking.

## 3. Discussion

This case represents a rare presentation of concurrent hepatic BML and AWE, raising strong suspicion for iatrogenic dissemination during prior gynecologic surgery.

The pathogenesis of BML remains unclear. However, several mechanisms have been proposed, including lymphovascular spread, coelomic metaplasia, metastasis from uterine leiomyosarcoma, and peritoneal seeding during uterine surgery [10]. On average, BML is often diagnosed 8.8–14.9 years after initial surgery for uterine leiomyoma [3, 10, 11]. In our patient’s case, the estrogen and PR positivity on biopsy of hepatic masses confirmed a gynecological tract origin. Additionally, her history of myomectomy, the 12‐year interval between primary surgery and diagnosis, and the widespread anatomical distribution of leiomyomas all support the theory of iatrogenic dissemination secondary to uncontained power morcellation.

It is also important to note that leiomyosarcoma was high on the differential given CT findings of massive heterogeneous hepatic masses, as the liver is a common site of metastasis [12, 13]. However, the patient’s clinical history, including her premenopausal status and prior myomectomy, along with the absence of the necrotic or cystic changes on imaging typically seen in leiomyosarcoma and confirmatory benign histopathology, ultimately ruled out this diagnosis [13, 14].

Similarly, there are multiple theories for the development of AWE, such as lymphovascular spread, coelomic metaplasia, and hormonal factors. However, iatrogenic implantation of endometrial cells remains the leading theory for pathogenesis, especially with a history of uterine surgery [6, 7, 15]. Endometrial tissue can be iatrogenically transplanted to the incision site in the abdominal wall, and endometrial cells proliferate overtime in response to hormonal stimulation, leading to the development of AWE [6, 7].

The coexistence of endometriosis and BML in this patient strongly suggests a shared pathogenesis related to prior laparoscopic surgery and iatrogenic dissemination of uterine tissue. These complications further underscore the importance of preventive strategies in gynecologic surgery. Alas, the FDA issued warnings in 2014 regarding power morcellation due to the risk of spreading occult malignancies and benign tissue like leiomyomas and endometriosis after our patient’s myomectomy in 2013. Power morcellation should be avoided, particularly in cases of uterine malignancy. When used, containment techniques should be prioritized to minimize the risk of iatrogenic dissemination and complications such as benign metastatic leiomyomatosis, parasitic leiomyomas, and endometriosis [16–18].

While surgical excision is the standard treatment for AWE, there are no standardized guidelines for the treatment of BML [6, 7, 10]. Surgical treatment is recommended, however, for localized and resectable lesions, as those who undergo complete tumor removal have favorable prognoses [19, 20]. In patients with estrogen and PR‐positive disease, oophorectomy may be considered to reduce hormonal stimulation and decrease the risk of recurrence, particularly in the presence of endometriosis. In this case, an ovary‐sparing hysterectomy was performed to avoid premature surgical menopause, given the patient’s young age as well as the presence of benign appearing ovaries during intraoperative inspection. Furthermore, no identifiable endometriosis was found in the pelvic and abdominal cavities intraoperatively, and the AWE was managed with wide local excision to minimize the risk of recurrence.

No routine postoperative medical therapy is established for BML or AWE [21]. However, given her ER/PR‐positive disease and ovary‐conserving surgical approach, medical hormonal therapy with gonadotropin‐releasing hormone agonists or aromatase inhibitors may be considered to prevent recurrence of BML, while also providing benefit in preventing recurrence of endometriosis [22, 23].

This case also highlights the importance of multidisciplinary collaboration, as the surgical management of this patient required coordination among surgical oncology, OB/GYN, and urology to address the extensive intraabdominal and pelvic disease. Furthermore, patients with a history of morcellated fibroid surgery may benefit from undergoing long‐term surveillance, especially when symptomatic or with new mass development, given the risk of recurrence and progression.

## Funding

The authors received no funding for this manuscript.

## Consent

No written consent has been obtained from the patient, as no patient‐identifiable data is included in the case report.

## Conflicts of Interest

The authors declare no conflicts of interest.

## Data Availability

The data that support the findings of this study are available upon request from the corresponding author. The data are not publicly available due to privacy or ethical restrictions.
